# MXene‐Integrated Responsive Hydrogel Microneedles for Oral Ulcers Healing

**DOI:** 10.1002/smmd.135

**Published:** 2025-02-26

**Authors:** Chuanhui Song, Minhui Lu, Ning Li, Hongcheng Gu, Minli Li, Ling Lu, Yu Wang

**Affiliations:** ^1^ Department of Oral and Maxillofacial Surgery Affiliated Hospital of Medical School Nanjing Stomatological Hospital Institute of Stomatology Nanjing University Nanjing China; ^2^ Department of Otolaryngology Head and Neck Surgery School of Biological Science and Medical Engineering Zhongda Hospital Southeast University Nanjing China; ^3^ Oujiang Laboratory (Zhejiang Lab for Regenerative Medicine, Vision and Brain Health) Wenzhou Institute University of Chinese Academy of Sciences Wenzhou China

**Keywords:** antibacterial, drug delivery, microneedle, MXene, oral ulcer

## Abstract

Glucocorticoids such as dexamethasone have shown promising therapeutic effects in conquering oral ulcers. Challenges in this area are focused on enhancing the localized curative effects and responsive release. Herein, we presented a novel MXene‐integrated responsive hydrogel microneedle delivering dexamethasone to promote the healing of oral ulceration. By loading MXene, the hydrogel microneedles enable NIR (Near Infrared)‐responsive release of the inner dexamethasone for inflammation control and tissue regeneration. In addition, the MXene‐induced local hyperthermia could inhibit the bacteria, preventing the possible infection of ulcer lesions in the oral cavity. Based on these features, we demonstrated that our strategy could relieve local inflammation, promote tissue reconstruction, and accelerate wound healing in rat oral ulcer models. Overall, these NIR‐responsive MXene‐integrated hydrogel microneedles show significant promise in promoting ulcer healing and bring new ways for oral disease treatment.

## Graphical abstract

The MXene‐integrated responsive hydrogel microneedles enable NIR‐responsive release of the inner dexamethasone for inflammation control and tissue regeneration in the oral ulcer site. In addition, the MXene‐induced local hyperthermia could inhibit the bacteria, preventing the possible infection for ulcer lesions and showing significant promise in promoting ulcer healing.

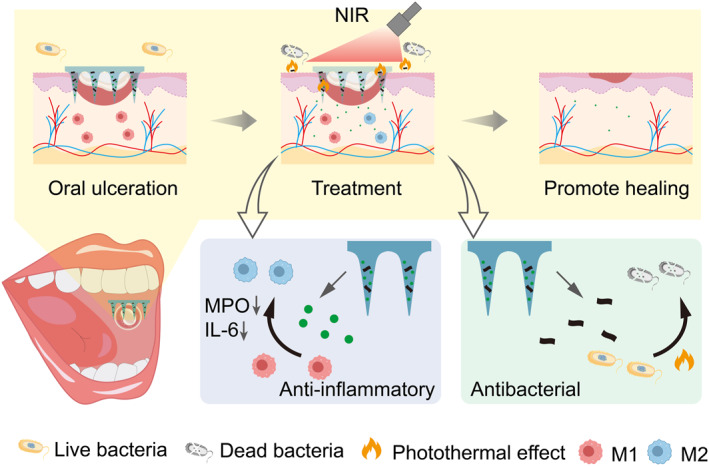

**Summary**

- The MXene‐integrated responsive hydrogel microneedles enable the NIR‐responsive release of the inner dexamethasone.
- The MXene‐induced local hyperthermia could inhibit the bacteria, preventing the possible infection.
- The novel strategy showed significant promise for inflammation control and tissue regeneration in the oral ulcer site.

## 1 Introduction

Oral ulceration is a common mucosal defect that impacts the oral cavity function as the pain and discomfort from the wound site [1, 2, 3, 4]. As a regular strategy, dexamethasone can modulate the immune microenvironment, exerting anti‐inflammatory effects and promoting oral ulcer healing [5, 6]. Oral administration is an easy way to get, while the side system effect dramatically hides the widely applied [7]. Formulations such as membranes, sprays, or tablets can act as alternative ways to local deliver medium [8, 9, 10, 11, 12]. However, the ways mentioned above ignore the controllable release profile of the integrated ingredient, leading to unsatisfactory effect. In addition, the persistently secreted salivary makes the delivery system hard to retain, and the inadequate concentration of the drug, which needs repeat give and unnecessary waste [13, 14, 15]. Therefore, it is highly desired to construct a local drug delivery system to achieve controllable release and long‐term retention to accelerate the healing of oral ulceration.

**FIGURE 1:**
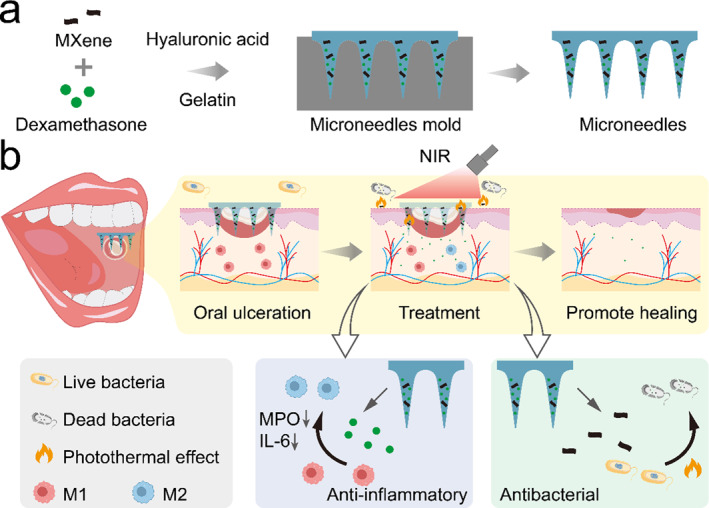
(a) The design and fabrication of the NIR‐responsive MXene‐integrated microneedles to promote oral ulcer healing. (b) The application of the microneedles in the oral ulcer site.

In this study, we developed a novel MXene‐integrated responsive hydrogel microneedle that delivers dexamethasone to promote the healing of oral ulceration, as schemed in Figure 1. Microneedles can penetrate the skin or mucosa painlessly and minimally invasively, showing potential application in local drug delivery [16, 17, 18, 19, 20]. Besides, after suitable modification, the microneedles have high drug‐carrying capacity and can be endowed with responsive release properties [21, 22, 23]. Thanks to these features, microneedles have been widely explored for drug delivery in different parts of the human body, such as the dermis, cardiology, uterus, and vasculature [24, 25, 26, 27, 28, 29, 30]. In contrast, MXene is a two‐dimensional material with excellent biocapacity and high photo‐heat convenience [31, 32, 33]. By loading in microneedles, the MXene can endow photothermal responsibility to achieve controllable release of inner ingredients, attracting much attention in biomedical [34, 35], while this NIR (Near Infrared)‐responsible microneedle remains to be explored in terms of dexamethasone delivery and oral ulcer management.

In this study, taking advantage of the NIR responsibility, we integrated MXene into microneedles to deliver dexamethasone into the oral ulcer site to accelerate ulcer wound healing. MXene was added to the microneedles consisting of hyaluronic acid and gelatin, forming microneedles. Upon NIR irradiation, the MXene‐based photothermal effect could induce the melt of the gelatin, accelerating the release of the inner dexamethasone. The released dexamethasone could relieve the inflammation reaction and accelerate the ulcer healing. Meanwhile, the hyperthermia from NIR could conquer porphyromonas gingivals (P.g.) to avoid the potential infection. Based on these attributes, it was demonstrated that this topical delivery system had favorable therapeutic performance on ulcer wounds in the rat model, suggesting that the novel microneedles could provide a promising option for oral ulceration and could apply in other stomatological disorders.

## 2 Results and Discussion

**FIGURE 2:**
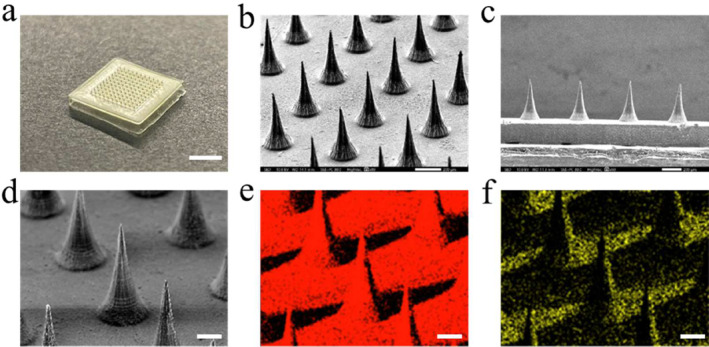
The characterization of the microneedles. (a) Digital picture of the resultant microneedles. Scale bar = 500 μm. (b) Scanning electron microscopy picture of the microneedles. Scale bar = 200 μm. (c) The lateral observation of the microneedles. Scale bar = 200 μm. (d–f) Elements analysis of the microneedles. Red: c; yellow: Ti. Scale bar = 100 μm.

To obtain the NIR‐responsive microneedle patches, gelatin and hyaluronic acid (HA) were mixed with MXene and dexamethasone. The PDSM (polydimethylsiloxane) model was designed to assist with the fabrication of the MNs. After dehydration, the resultant microneedles with translucence were obtained (Figure 2a). To further observe the microstructure of the proposed MNs, scanning electron microscope and fluorescence microscope were used. As shown in the Figure 2b and Supporting Information S1: Figure S1, the tips were uniform and regular (Figure 2b). The images showed that the length of the microneedle's tips was about 450 μm, and the diameters were about 200 μm (Figure 2c). To investigate the MXene‐loaded profile of the microneedles, an energy‐dispersive X‐ray spectroscope was conducted to analyze the Ti distribution (Figure 2d). The above results showed that the Ti elements were dispersed uniformly in the matrix of the microneedles (Figure 2e and f). All those results proved that the MXene‐integrated MNs were conducted successfully.

**FIGURE 3:**
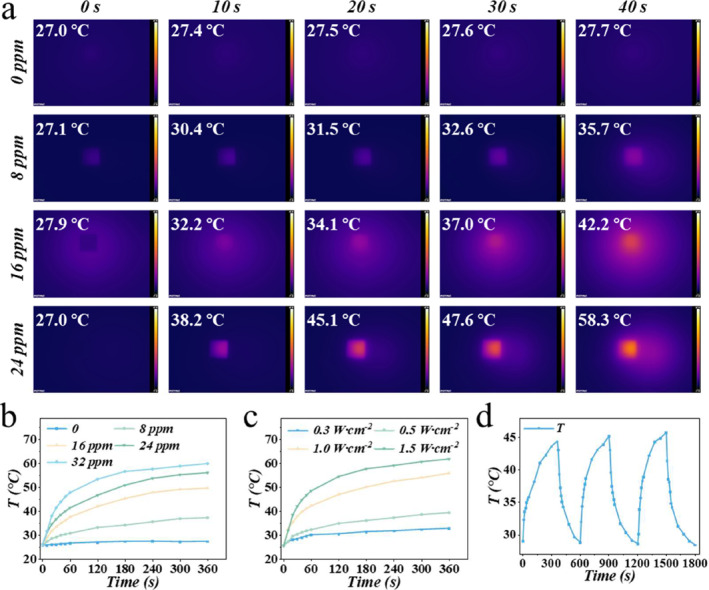
The photothermal effect of the microneedles. (a) The thermal images of the microneedles with different concentrations of MXene upon NIR application. (b) The temperature change of the microneedles with different MXene concentrations. T: temperature. (c) The temperature change profile of the microneedles depends on different NIR power intensities. T: temperature. (d) The temperature change of the microneedles with circled irradiation.

As a novel 2D photo‐responsive material, MXene has been widely used in biomedical applications for many years. Based on the photo‐heat conversion of the MXene, we further investigated the photothermal conversion of the resultant microneedles. The temperature of the MNs with different concentrations of MXene increased upon the NIR irradiation (Figure 3a and b). With 40 s of irradiation, the temperature of the microneedles could reach 58°C, possessing potency in the photothermal application in the antibacterial. Also, the temperature evaluation of the MXene‐integrated microneedles was correlated with the power intensity of the NIR, offering flexible regulation when used in the biomedical field (Figure 3c). The stability of photothermal conversion is also a critical evaluation index of the application of the photothermal agents. After three cycles of warming and cooling, the fabricated microneedles were still able to maintain the heating performance of the temperature, indicating that they have good photothermal stability (Figure 3d).

**FIGURE 4:**
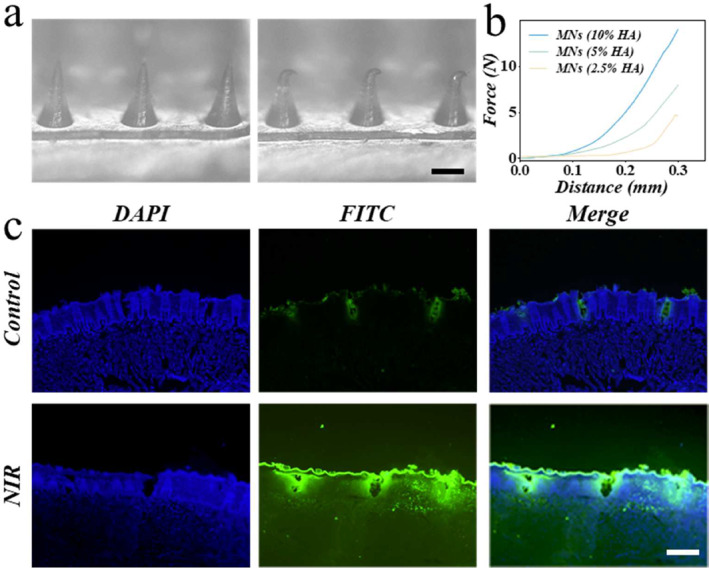
The mechanical properties and drug‐release properties of the microneedles. (a) The digital images of the microneedles before and after the mechanical test. (b) The force profile of the microneedles from the different concentrations of the HA. (c) The fluorescent images of the tissue with/without NIR application. Scale bar = 100 μm.

To prepare the application of the microneedles, their mechanical properties and drug release specialties were evaluated. Pressure testing tested the pressure‐displacement curves of microneedles of different compositions (Figure 4a). The results showed that with the increase in the concentration of the HA, the mechanical strength was increased (Figure 4b). Considering the penetration of the oral mucosa and the ease of preparation, moderate concentrations were selected as the final microneedle‐making formulation. To evaluate the bio‐safety of the microneedles, the 3T3 and HUVEC cell lines were co‐cultured with the microneedles. The living cells were stained with calcein acetoxymethyl ester (Supporting Information S1: Figure S2), which showed that the microneedles owned wonderful bio‐safety. Based on the photothermal effect and the cell experiment, the final concentrations of the MXene and dexamethasone were determined to fabricate the microneedles. To assess the NIR‐responsive ability of the microneedles, the fluorescent dye‐integrated microneedles were inserted into an ex vivo tissue. After NIR irradiation, the tissue was cut into slices and observed under a fluorescence microscope. After NIR application, the fluorescence diffusion is much larger, suggesting that there is much more drug release (Figure 4c). Also, as demonstrated by the quantitative data, the length, width, and average intensity of the fluorescent regions are significantly enhanced after NIR irradiation (Supporting Information S1: Figure S3), which is due to the fact that high temperatures cause the gelatin to melt, which in turn accelerates the release of the drug in a hyperthermia environment.

**FIGURE 5:**
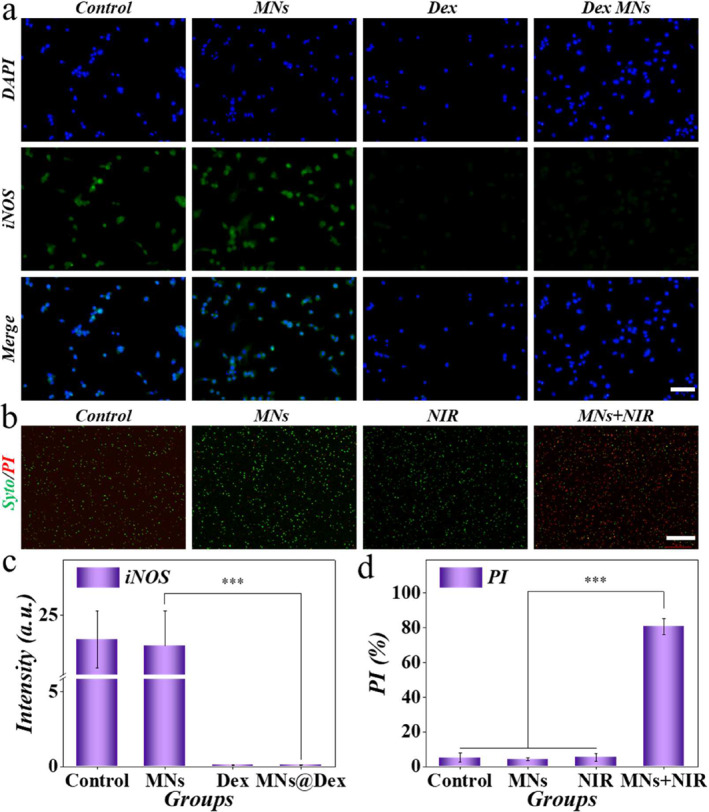
The anti‐inflammation and anti‐bacterial effect of the microneedles. (a) The iNOS staining of the RAW264.7 after different treatments. Dex, dexamethasone; MNs, microneedles. Scale bar = 100 μm. (b) Live/dead fluorescent staining of the P.g. in various groups. Scale bar = 100 μm. (c) Quantitative data of (a). (d) Quantitative data of (b).

The inflammatory response at the ulcer can inhibit tissue rebuilding, leading to slow wound healing. To test the mitigating effect of drug‐loaded microneedles on inflammation, we utilized induced macrophages as model cells. After being treated with different conditions, the dexamethasone‐loaded microneedles demonstrated similar inflammation suppression effects as the drug (Figure 5a). The dexamethasone and dexamethasone‐loaded microneedles both downregulated the expression of iNOS, representative markers of type I macrophages (Figure 5c). As the dexamethasone was directly added to the medium, the effect may be better than that of the microneedle group. The oral cavity is a complex environment with a wide range of bacteria, and the prevention of bacterial infections is also a property that biomaterials need to possess. P.g. (porphyromonas gingivals) was co‐cultured with the dexamethasone‐loaded microneedles. Upon NIR application, the proportion of dead bacteria significantly increased compared with the other groups (Figure 5b and d). MXene can induce local hyperthermia from the localized surface plasmonic resonance effect, showing huge potential in antibacterial applications [36]. All those results indicated that the microneedles could inhibit the excessive inflammatory response and prevent possible bacterial infections, facilitating the healing of oral ulcer wounds.

**FIGURE 6:**
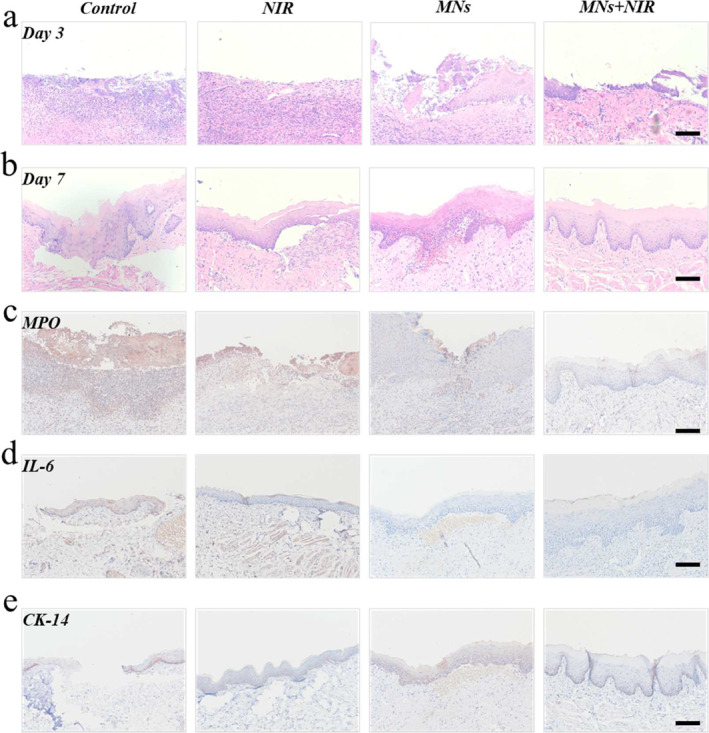
The pathological staining of the tissue from the ulcer animal. (a, b) The HE staining of the ulcer tissue in different groups at Day 3 and 7. (c) The MPO staining of the tissue. (d) The IL‐6 staining of the tissue after treatments. (e) The CK‐14 staining of the tissue in different groups. Scale bar = 100 μm.

Encouraging by the in vitro results, an oral ulcer wound model was established in rats to evaluate the in vivo effects. After the ulcer wound formation, the animals were divided into four groups following the wound observation. To test the inflammation response and the tissue healing, the tissue was collected on the third day (acute inflammation) and the seventh day (tissue regeneration). The microneedles with the NIR group showed wound healing on the third day, and after 7 days, the local tissue was repaired completely (Figure 6a and b). Neutrophils are characteristic cells of the acute inflammatory phase, and myeloperoxidase is a representative marker. Figure 6c (Day 3) showed that the expression of the myeloperoxidase was relatively high in the control group, while the microneedles with NIR could inhibit the expression (Supporting Information S1: Figure S4a). Also, Interleukin 6 represents the level of inflammation within localized tissues, and immunohistochemical staining at day 7 confirmed that therapeutic strategies can lead to significant decreases (Figure 6d and Supporting Information S1: Figure S4b). The continuity of cytokeratin 14 represented the integrity of epithelial repair, indicating that the treatment group was able to promote ulcer healing well (Figure 6e and Supporting Information S1: Figure S4c). The HE staining of the organ from the rats after experiments showed no obvious change, demonstrating the biocompatibility of the strategy (Supporting Information S1: Figure S5).

## 3 Conclusion

In summary, we have developed MXene‐integrated microneedles to deliver dexamethasone to promote oral ulcer wound healing, with the ability of inflammation relief and regeneration facilitated. Compared to traditional strategies like patches and membranes, the microneedles can enhance local retention, maintaining locally high drug concentrations. Also, benefiting the penetration of the needles, the microneedles can deliver the ingredients into the deep tissue with minimal damage instead of just affecting the surface of the tissue. Although local injections also allow for deep‐tissue drug delivery, the accompanying pain and fear are still not readily accepted by patients with oral disease. The lack of a designed drug delivery system, moreover, is not characterized by drug‐responsive release, making the drug uncontrollably released. Thus, it is highly desired to develop well‐designed methods that better meet the complicated needs of oral disease wounds.

Herein, we have fabricated NIR‐responsive hydrogel microneedles with the capacity of dexamethasone to be controllably and responsively released inside the tissue to provide an inspiring strategy for oral ulcer healing. The MXene endowed the resultant microneedles with NIR responsive property as the photo‐heat conversion effect. Upon NIR irradiation, the subsequent hyperthermia could melt the gelatin, facilitating the tissue penetration of the integrated dexamethasone. The released drug can relieve the local inflammation and promote tissue regeneration. Furthermore, the MXene‐induced hyperthermia could inhibit bacteria, preventing the possible bacterial infection in the oral cavity. It was proved that when applied in a rat model with oral ulcer wounds, the designed responsive hydrogel microneedles could promote wound healing and inhibit local inflammation. These features endow the new microneedles with great potential for tissue repair and regenerative medicine in oral disease.

## 4 Experimental Section/Methods

### 4.1 Materials

Gelatin was obtained from the sigma. MXene was purchased from XFNANO. The HA, dexamethasone, cell culture, DAPI, and fetal bovine serum were from Adamas‐beta. MPO, IL‐6, CK‐14 antibody and CoraLite 488 conjugated IgG (H + L) (SA00013‐2) were obtained from Proteintech.

### 4.2 Preparation of Microneedles

The microneedles were fabricated with a micromold. The mixed solution (gelatin, HA, MXene, and dexamethasone) was added to the mold. After centrifugation, the bubbles were removed, and the mold‐containing solution was kept at room temperature for 24 h to remove the excess water. A series of concentrations of gelatin and HA were prepared to choose the most suitable concentration.

### 4.3 Characterization

The dried microneedles were placed on the microscope stage. After the gold spraying, the samples were observed with the scanning electron scope. The analysis of the elements was conducted during the observation.

### 4.4 Evaluation of Photothermal Property

The microneedles with different concentrations of MXene (0, 8, 16, 24, 32 ppm) were prepared to detect the temperature upon the NIR irradiation. Also, the different power intensities of NIR were used to evaluate the temperature change of the microneedles. During the experiments, the microneedles without MXene were selected as the control group, and the temperature recorder was used to detect the temperature of the microneedles.

### 4.5 Evaluation of the Mechanical Properties

Different concentrations of HA (10%, 5%, 2.5%) were chosen to make microneedles. The gelatin concentration was 40%. The force sensor was used to measure the force of the tips upon the microneedles.

### 4.6 Drug Loading and Release Measurement

To assess the drug loading of the microneedles, the FITC was added to the solution. After drying, fluorescence microscopy was used to observe the microneedles. To evaluate the NIR‐responsive property of the microneedles, the mouse tissue after microneedle application was fixed cut to observe the fluorescence distribution. The area of the fluorescence was measured.

### 4.7 The Anti‐Inflammation of the Microneedles

The LPS‐induced macrophages were used to assess the anti‐inflammation of the microneedles. The LPS (500 ng/mL) was added to the RAW 264.7 for 24 h. Microneedles with/without dexamethasone were applied to the cell culture medium. After co‐culturing for 24 h, the cells were collected, fixed, and stained to observe the iNOS (inducible nitric oxide synthase, M1 marker).

### 4.8 The Antibacterial of the Microneedles

P.g. was used to detect the antibacterial effect of the microneedles. The microneedles were co‐cultured with P.g. after NIR application, the culture medium was collected. The bacteria were resuspended with PBS to 200 μL, and 1 μL syto and 5 μL PI were added for 30 min.

### 4.9 Animal Experiments

The female Sprague‐Dawley (180–220 g) rats were purchased from the Huachuang Sino. The Animal Investigation Ethics Committee of The Affiliated Drum Tower Hospital of Nanjing University Medical School reviewed all animals' experimental protocols and care (2023AE02009). To establish the oral ulcer model, a 5 mm diameter filter paper soaked in 70% acetic acid was applied to the buccal region of the rat's mouth for 3 min. The buccal region was wiped dry of saliva to prevent dilution of the acetic acid. After 2 days, the ulcer could be observed in the oral cavity. Microneedles were applied to cover the wound (MXene: 24 ppm; dexamethasone: 10 mg/mL in the mixed solution). At the days 3 and 7, the tissue was collected for pathological staining (HE, MPO, IL‐6 and CK‐14). At the end of the treatment cycle, major organs are collected for pathological testing to evaluate the safety of the organism in vivo.

### 4.10 Statistical Analysis

All of the data analyses in this study were conducted using Origin. Data in the graphs and tables are presented as mean ± standard deviation. Statistical analyses were made using Student's t‐test (two groups) or one‐way ANOVA followed by Tukey's test (over two groups). A value of *p* < 0.05 was regarded as statistically significant.

## Author Contributions

L.L. and Y.W. conceived the idea. C.H.S. conducted experiments and data analysis. C.H.S. and M.H.L. wrote the manuscript. N.L. assisted with paper writing. H.C.G. and M.L.L. assisted with revision.

## Ethics Statement

The Animal Investigation Ethics Committee of The Affiliated Drum Tower Hospital of Nanjing University Medical School reviewed all animals' experimental protocols and care (2023AE02009).

## Conflicts of Interest

The authors declare no conflicts of interest.

## Supporting information

Supporting Information S1
