# Supplementary material for: MXene‐Integrated Responsive Hydrogel Microneedles for Oral Ulcers Healing
Source: Smart Med. 2025 Feb 26;4(1):e135. doi: 10.1002/smmd.135 (PMC11862566; doi:10.1002/smmd.135)
Supplement: Supplementary file 1 — Supporting Information S1 [file SMMD-4-e135-s001.docx]

Supporting information

**MXene-integrated responsive hydrogel microneedles for oral ulcers healing**

*Chuanhui Song, Minhui Lu, Ning Li, Hongcheng Gu*, Minli Li*, Ling Lu*, Yu Wang **


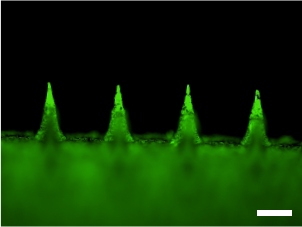


Figure S1. The fluorescent image of the microneedles. Scale bar=200μm.


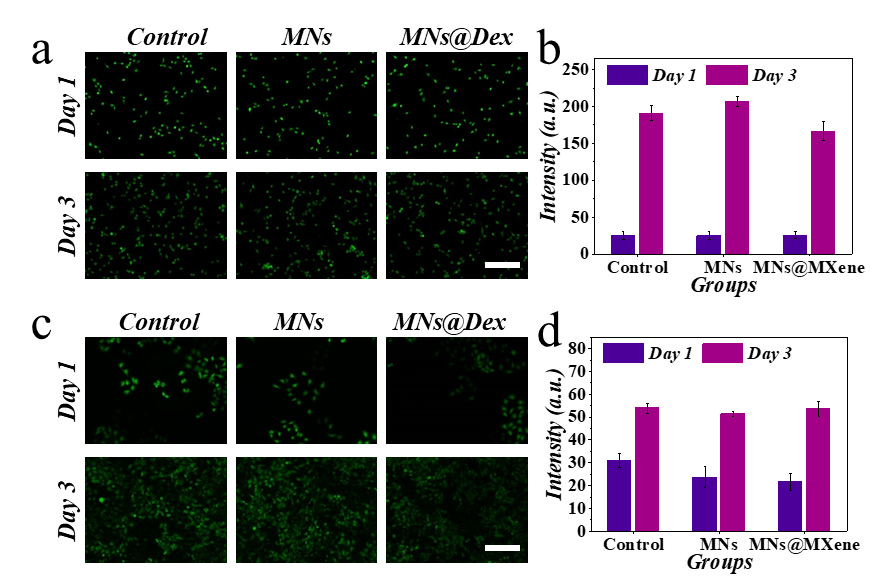


Figure S2. The (a) live staining of the 3T3 cells and the (b) quantitate data. The (c) live staining of the HUVEC cells and the (d) quantitate data


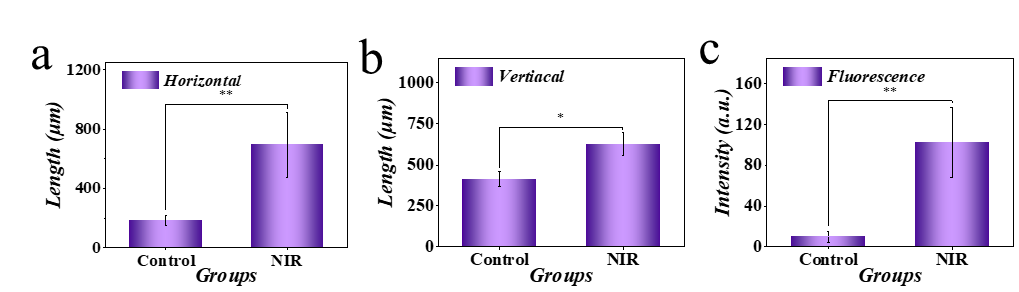


Figure S3. The quantitative analysis of the fluorescent (a) horizontal length, (b) vertical length and (c) intensity.


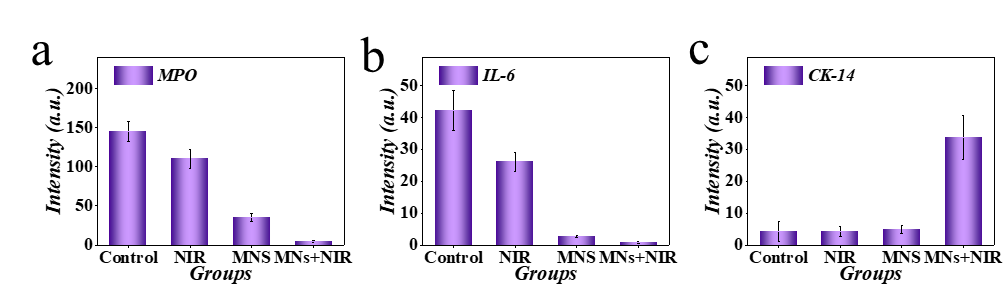


Figure S4. The quantitative analysis of the Figure. 6 c,d,e.


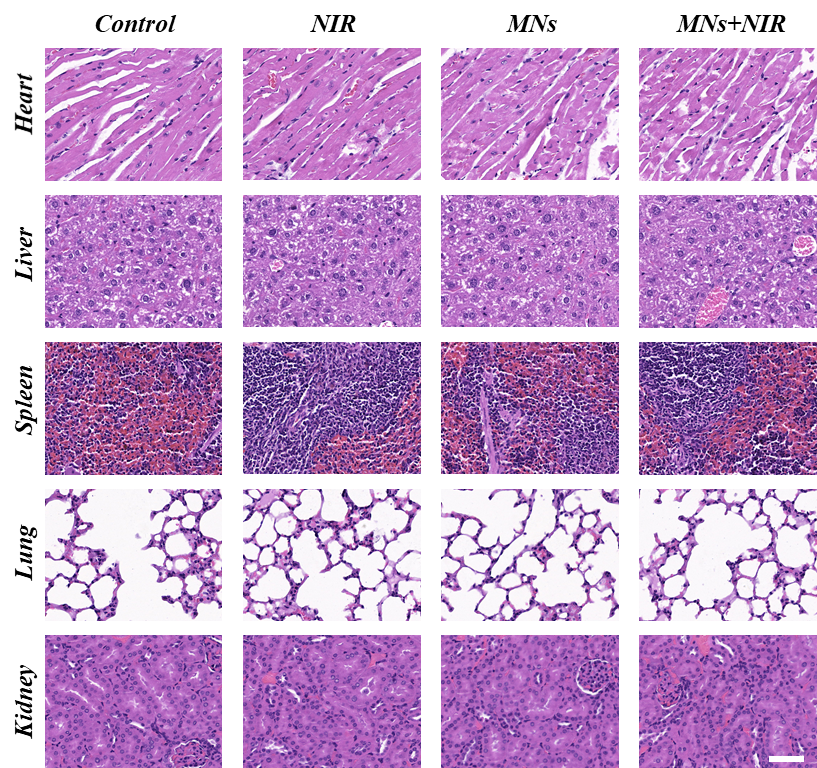


Figure S5. The HE staining of the organ from the rats at the end of the treatments. Scale bar=100μm.
